# Identification of a Quality Marker (Q-Marker) of Danhong Injection by the Zebrafish Thrombosis Model

**DOI:** 10.3390/molecules22091443

**Published:** 2017-08-31

**Authors:** Yuqing Qi, Xiaoping Zhao, Hao Liu, Yimin Wang, Chao Zhao, Tao Zhao, Buchang Zhao, Yi Wang

**Affiliations:** 1 College of Pharmaceutical Sciences, Zhejiang University, Hangzhou 310058, China; qiwei0571@163.com (Y.Q.); liuhao922@zju.edu.cn (H.L.); 2 College of Preclinical Medicine, Zhejiang Chinese Medical University, Hangzhou 310053, China; 3 Shandong Buchang Pharmaceutical Co., Ltd., Heze 274000, China; qianke5925@163.com (Y.W.); zbtzhaotao@163.com (C.Z.); zbtzhaochao@163.com (T.Z.); shiyang494118816@163.com (B.Z.)

**Keywords:** Chinese medicine, batch-to-batch consistency, zebrafish thrombosis model, rosmarinic acid, p-coumaric acid

## Abstract

Quality-marker (Q-marker) is an emerging concept to ensure the quality and batch-to-batch consistency of Chinese medicine (CM). However, significant difficulties remain in the identification of Q-markers due to the unclear relationship between complex chemical compositions and the pharmacological efficacy of CM. In the present study, we proposed a novel strategy to identify the potential Q-marker of danhong injection (DHI) by an in vivo zebrafish thrombosis model. The anti-thrombotic effects of DHI and its major constituents were evaluated by the zebrafish model of arachidonic acid (AA)-induced thrombosis. The results indicated that DHI can attenuate tail venous thrombus and recover the decrease of heart red blood cell (RBC) intensity in a dose-dependent manner. The result that DHI prevented the formulation of thrombosis in zebrafish was also validated in the zebrafish thrombosis model with green fluorescence protein (GFP)-labeled hemoglobin. The major components of DHI, namely danshen (DS) and honghua (HH), as well as the major chemical constituents of DHI, also exerted anti-thrombotic effects, among which rosmarinic acid (RA) and p-coumaric acid (pCA) showed moderate anti-thrombotic effects. This is the first time that pCA from HH has been found as an active compound exerting an anti-thrombotic effect in a dose-dependent manner, whose IC_50_ value is approximately 147 μg/mL. By analyzing 10 batches of normal DHI samples and five abnormal samples by high-performance liquid chromatography (HPLC), we found the contents of pCA and RA can be positively correlated to the anti-thrombotic effect of DHI, suggesting that pCA and RA could be potential Q-markers of DHI to ensure batch-to-batch consistency. Our findings illustrated that discovering major active compounds from CM by in vivo pharmacological models can be a useful approach to identifying Q-markers of CM, and in vivo pharmacological models can be a potential tool to evaluate batch-to-batch consistency of CMs.

## 1. Introduction

Quality assessment of Chinese medicines (CMs) is an important issue in the modernization of traditional Chinese medicine (TCM), for CMs have been widely applied in clinical therapy in China and other developing countries [1,2]. Various analytical methods have been adopted to ensure the quality of CM products by measuring the concentration of major chemical ingredients by high performance liquid chromatography (HPLC), gas chromatography (GC), liquid chromatography-mass spectrometry (LC-MS), near-infrared spectroscopy (NIRS), and so on [3]. Chen [4] established a method for the characterization of multi-constituents and applied it for the quality control of PSORI CM01, a TCM preparation. Chromatographic fingerprinting [5] was adopted to ensure batch-to-batch consistency of CM. Xie et al. [6] developed an approach to evaluate the quality consistency of liuwei dihuang pills produced by different manufacturers based on HPLC-fingerprint and chemometric methods.

In spite of a large number of successes that have been achieved in the past decade, the selection criteria of chemical entities for quality assessment still remains uncertain because it lacks sufficient scientific evidence to indicate the relationship between the concentration of chemical markers and clinical efficacy. Thus, it is suggested that biological efficacy should be under consideration in quality control in the renewed version of the Botanical Drug Development Guideline for Industry proposed by the Food and Drug Administration (FDA) [7]. Recently, several attempts have been taken to employ biological activity assays for the quality control of CM products. Xiang [8] combined a metabolomics study with quality control based on gas chromatography-mass spectrometry (GC-MS) and successfully distinguished the samples from different species and ecotypes. Liu [9] proposed that the intestinal permeability assay can be utilized for quality and safety control since intestinal absorption is one of the most important issues for the pharmacokinetic properties of CMs.

Recently, the concept of the quality marker (Q-marker) has been proposed by Liu [10]. Q-marker refers to a series of intrinsic or processing/preparation-resultant compounds contained in CMs and their products, and is closely associated with the efficacy and safety of CMs [11]. Thus, the efficacy-based quality assessment could be realized using the content analysis of Q-markers. Therefore, methods which can distinguish the efficacy-representative compounds of a specific disease model for discovering Q-markers of CMs are urgently needed.

As a versatile and flexible model organism, zebrafish (*Danio rerio*) have been widely employed for biological and pathological studies, such as toxicity research [12] and pathological mechanism research [13]. Compared with the model organisms such as mice [14], rats [15], and *Drosophila melanogaster* [16,17,18], the zebrafish model organism has gathered a large number of advantages, including the optical clarity of the embryos and the ease for genetic manipulation, as well as the genetic similarity with humans [19]. Zebrafish was thought to be more appropriate for the research of angiocardiopathy than other models, due to the remarkable similarity of its cardiovascular system to humans [20] and the operation convenience. On the basis of those attributes, zebrafish provide opportunities to accelerate the drug discovery process, including target identification, disease modeling, lead discovery, and toxicology [21]. However, to the best of our knowledge, there is no report to apply zebrafish models to establish an efficacy-based quality assessment approach to CM.

Danhong injection (DHI) is one of the most-sold CMs in China, which is extensively used to treat cardiovascular and cerebrovascular diseases in the clinic [22]. It consists of rhizoma salviae miltiorrhizae (*Salvia miltiorrhiza* Bge., Labiatae, danshen in Chinese) and Flos Carthami (*Carthamus tinctorius* L., Compositae, *Salvia militiorrhiza* Bunge, honghua in Chinese) components and contains various chemical constituents, such as salvianolic acid B (SaB), danshensu (DSS), lithospermic acid (LA), and rosmarinic acid (RA) [22]. Both danshen (DS) and honghua (HH) are widely used herbs. DS is the dried root of *Salvia miltiorrhiza* and has been applied for treating cardiovascular and cerebrovascular diseases in China and other countries, such as Japan and the United States [23]. HH is the flower of an annual or biennial herbal plant [24], and has been employed for the treatment of thrombotic disorders by activating the blood circulation to mitigate blood stasis, according to the traditional Chinese medicine theory [25]. The criterion of quality assessment for DHI in the Pharmacopoeia of the People’s Republic of China owed the quality of DHI to five compounds, i.e., SaB, DSS, RA, protocatechuic aldehyde (PA), and p-coumaric acid (pCA). However, there is still a lack of sufficient scientific evidence to illustrate the relationship between the content of those compounds and the efficacy, since current studies regarding the quality assessment of DHI have mainly focused on the content assessment [26]. The present study developed a novel approach to identify Q-markers by evaluating the anti-thrombotic effect of major constituents of DHI via an in vivo zebrafish model. Furthermore, it was applied to distinguish the abnormal samples from normal batches and to evaluate the batch-to-batch consistency of DHI.

## 2. Results

### 2.1. The Development of the Zebrafish Thrombosis Model

**Figure 1:**
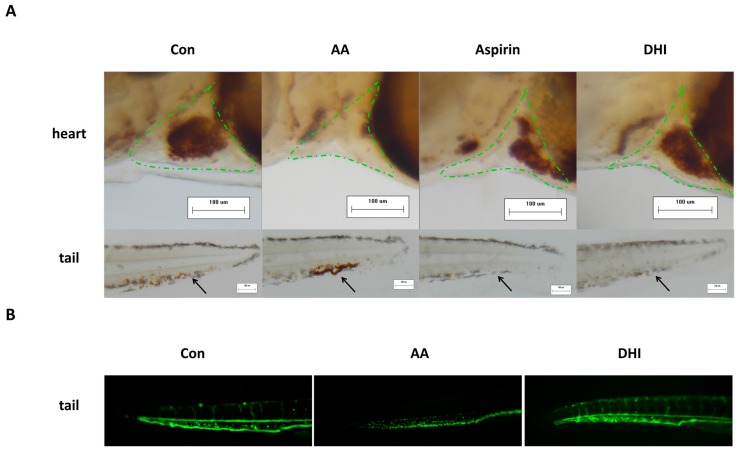
(**A**) Heart red blood cells (green dashed line) and tail venous thrombus (black arrows) stained with *o*-dianisidine in the zebrafish of the control group, AA group (AA 40 μM), positive drug group (aspirin 22.5 μg/mL), and DHI (100 μL/mL) group, at a 100× magnification; (**B**) Tail venous thrombus in the transgenic LCR-GFP zebrafish of the control group, model group (AA 40 μM), and DHI (100 μL/mL) group, at a 100× magnification. Con, AA, DHI and LCR-GFP stand for control, arachidonic acid, danhong injection, and locals control region-green fluorescence protein respectively.

The zebrafish thrombosis model was established by exposing the zebrafish to 40 μM arachidonic acid (AA) for one hour. The representative images of zebrafish stained by *o*-dianisidine were captured by a stereomicroscope. As shown in Figure 1A, compared with the control, it is clear that visible red blood cells (RBCs) almost disappeared in the heart of AA-treated zebrafish (marked by the green dashed line), whilst the accumulation of RBCs in the vessel at the tail part of zebrafish (as marked by the black arrow) was increased after AA treatment. However, treatment of aspirin at 22.5 μg/mL could obviously recover the quantity of heart RBCs, which is in accordance with previous reports [27]. Figure 1A also showed that DHI could significantly inhibit the tail venous thrombus and recover the quantity of heart RBCs in the zebrafish thrombosis model for the first time, which suggested that DHI can exert an anti-thrombotic effect in vivo. The effect of DHI against AA-induced thrombosis was further validated in the transgenic LCR-GFP (locals control region-green fluorescence protein) zebrafish [28] whose hemoglobin was GFP-labeled. As shown in Figure 1B, the AA-treated zebrafish exhibited dark and discontinuous blood flow compared to the continuous and bright flow of the control, which could be recovered by DHI.

**Figure 2:**
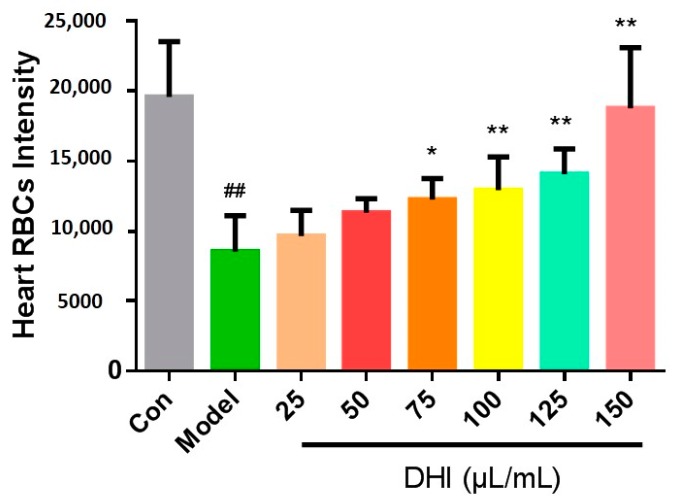
Heart red blood cells intensity in the zebrafish of control group, model group (AA 40 μM), and DHI group of different concentrations (25–150 μL/mL). The error bars represent the standard deviation based on ten repeated samples. All data was represented by the mean ± SD, ^##^ *p* < 0.01, ** *p* < 0.01, * *p* < 0.05, *n* = 10.

The effects of DHI against AA-induced thrombosis in zebrafish were quantitatively assessed by measuring RBC intensity of the heart area. The results showed that pre-incubation of DHI in doses of 25, 50, 75, 100, 125, and 150 μL/mL could recover the heart red blood cells in a dose-dependent manner (Figure 2). When treating zebrafish by DHI with a 75 μL/mL or higher dosage, it significantly recovered the heart RBC intensity compared to zebrafish with AA-induced thrombosis. All those findings above suggested the thrombosis model was appropriate for the efficacy assessment of DHI.

### 2.2. The Constituents of DHI Improved the Zebrafish Thrombosis

**Figure 3:**
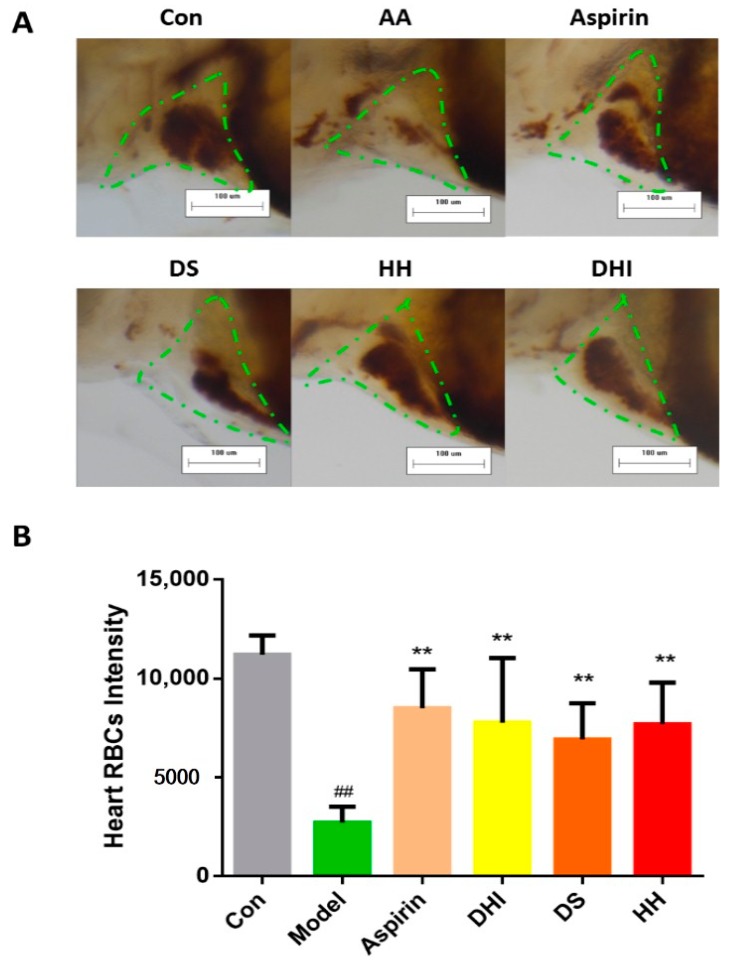
Heart red blood cells (green dashed line) stained with *o*-dianisidine (**A**) and the heart red blood cell intensity in the zebrafish of the control group, model group (AA 40 μM), positive drug group (aspirin 22.5 μg/mL), danshen (DS) group (100 μL/mL), honghua (HH) group (100 μL/mL), and danhong group (100 μL/mL), at a 100× magnification; (**B**) The error bars represent the standard deviation based on ten repeated samples. All data was represented by the mean ± SD, ^##^ *p* < 0.01, ** *p* < 0.01, *n* = 10.

The effects of the two major components of DHI, named danshen (DS) and honghua (HH), on the thrombosis of zebrafish was also evaluated by the model to further illustrate which is the active component of DHI. DS, HH, and DHI were given at the concentration of 100 μL/mL, and aspirin was given at 22.5 μg/mL as the positive drug. As shown in Figure 3A, the heart area was circled by the green dashed line. The heart RBC intensity of zebrafish protected by DS, HH, and DHI significantly increased compared to the AA-treated zebrafish, respectively. The quantitative results are shown in Figure 3B, indicating that DS and HH could both significantly improve the thrombosis similarly to DHI.

### 2.3. The Identification of Active Compounds in DHI on Anti-Thrombosis Effects

**Figure 4:**
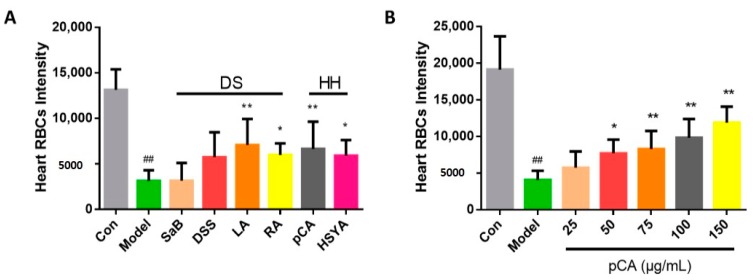
Heart red blood cells intensity in the zebrafish of the control group, model group (AA 40 μM), and compounds of DHI groups (100 μg/mL) (**A**) and the p-coumaric acid levels of different concentration groups; (**B**) The error bars represent standard deviation based on ten repeated samples. All data was represented by the mean ± SD, ^##^ *p* < 0.01, ** *p* < 0.01, * *p* < 0.05 (*n* = 10). SaB, DSS, LA, RA, pCA and HSYA stand for salvianolic acid B, danshensu, lithospermic acid, rosmarinic acid, p-coumaric acid and hydroxysafflor yellow A, respectively.

The effects of several compounds [29] in DHI were also evaluated on the model to identify the active chemicals which could be candidates for Q-markers of DHI. Four chemicals belonged to DS, including SaB, DSS, LA, RA, and two chemicals belonging to HH, including pCA and hydroxysafflor yellow A (HSYA), were evaluated. As the statistical results showed in Figure 4A, the LA, RA, pCA and HSYA proved to be significantly effective on this model at the concentration of 100 μg/mL. Furthermore, LA and pCA produced more significant effects than RA and HSYA, in which the pCA was reported for the first time. Therefore, to further confirm the effective concentration of pCA on this model, we treated this model with pCA at concentrations of 25, 50, 75, 100, and 150 μg/mL, and it suggested that the effects of pCA were dose-dependent in the range of 25–150 μg/mL, and became effective at a 50 μg/mL or higher dosage, and exhibited a processed IC_50_ value of 147 μg/mL.

### 2.4. Identification of Q-Markers and the Application to Ensure Batch-To-Batch Consistency of DHI

**Figure 5:**
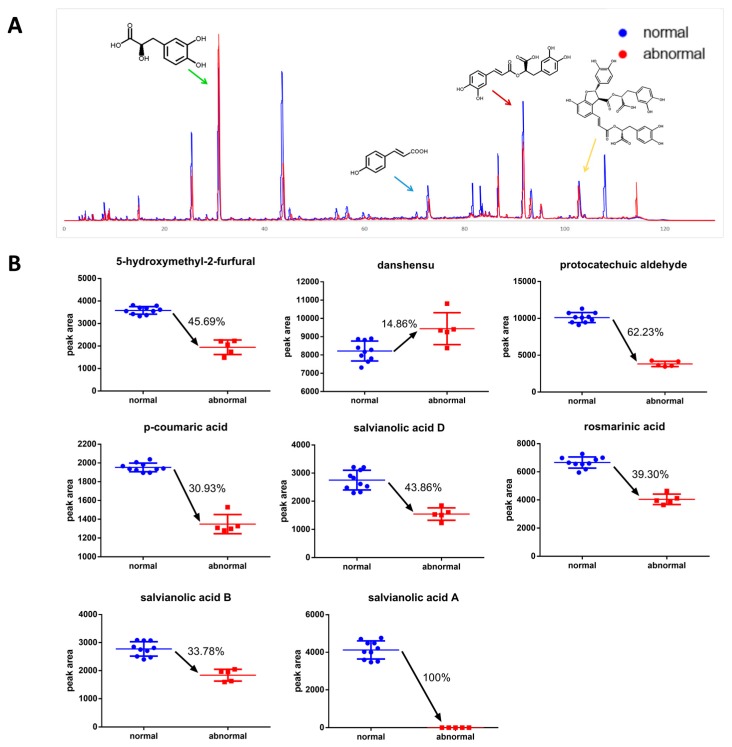
The distinction of the HPLC chromatogram (**A**) and compound content (**B**) between normal and abnormal DHIs.

**Figure 6:**
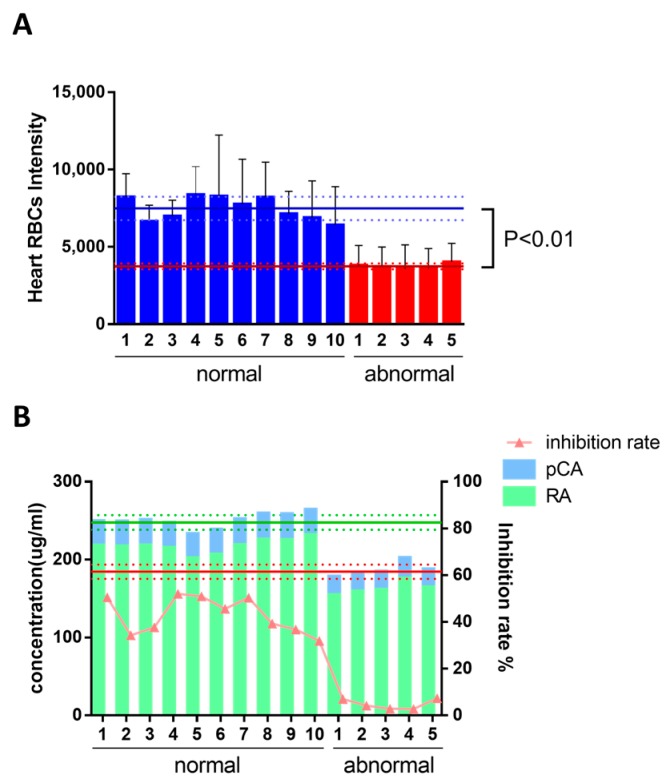
Heart red blood cell intensity in the zebrafish of normal and abnormal DHI (**A**) The distinction of the total concentration of RA and pCA in accordance with the inhibition rate between the normal and abnormal DHI; (**B**) The error bars represent the standard deviation based on ten repeated samples. Data in Figure 6A was represented by the mean ± SD and data in Figure 6B was represented by the mean, *n* = 10.

The differences of efficacy and compound content between normal and abnormal DHI were evaluated by the model and HPLC, respectively, in order to decide whether the model was suitable to ensure batch-to-batch consistency, and to screen the chemicals that could be regarded as Q-markers as well. There is significant distinction of the compound content between normal DHI and abnormal ones according to the analysis by HPLC, as the chromatogram showed in Figure 5A. The peaks of qualitatively analyzed compounds, namely DSS, pCA, RA, and SaB, were marked by green, blue, red, and yellow arrows, respectively, and the structural formulas were given beside the peaks. The content changes of major compounds in DHI are shown in Figure 5B. The content of major compounds DSS, SaB, RA, and pCA are 1293.17 ± 59.19, 305.21 ± 18.62, 215.17 ± 8.84, and 32.53 ± 0.79 μg/mL in DHI, respectively, according to the results of quantitative analysis by HPLC. Furthermore, the correlation coefficients between the anti-thrombotic effect and the contents for major compounds in DHI were calculated, as shown in Table 1. It is clear that all of the contents present a positive correlation except DSS, which is the degradation product of salvianolic acid B. As shown in Figure 6A, the efficacy between normal DHI and abnormal samples proved to be significantly distinctive by a *t*-test (the mean and mean ± SD of heart RBC intensity were marked in Figure 6A with blue and red lines, representing normal DHI and abnormal samples, respectively), which is in accordance with the content analysis shown in Figure 5. Based on the active compounds determined by the zebrafish thrombosis models and the results of the content analysis by HPLC, two representative potential Q-markers could be determined as RA and pCA, and the total content of RA and pCA was also proved to be significantly distinctive (Figure 6B, the mean and mean ± SD of total content were marked in the figure with green and red lines, representing normal DHI and abnormal samples, respectively), in accordance with the efficacy of normal and abnormal DHI, suggesting that the RA and pCA could be regarded as the potential Q-markers of DHI in quality assessment.

## 3. Discussion

The quality assessment of CM has become an important issue in recent years. In spite of great achievements, current approaches for CM quality assessments have always concentrated on the method of content analysis, which might be unsuitable for CM since CM is a complex multi-component system [10]. The present study put forward a method based on the efficacy of CM to ensure batch-to-batch consistency and identified efficacy-based Q-markers for the content analysis of quality assessment.

Zebrafish have been employed as model organisms for decades [30], and now are used mainly in the aspect of physiology [31], pharmacological studies [32], and chemical toxicity investigation [33]. However, its value in the quality assessment of CM has not been extensively explored and exploited.

The present study intended to identify bio-active Q-markers for the quality assessment of CM through a zebrafish model, and to test whether the model is appropriate for the batch-to-batch consistency assurance of CM. As for DHI, it was reported that it could reduce the inflammatory factor hsCRP (high sensitive C-reactive protein), IL-6 (inter-leukin 6), MCP-1 (monocyte chemoattractant protein-1), and TXB_2_ (thromboxane B_2_) levels, so as to prevent the occurrence of thrombosis and the aggregation of platelets [34]. Therefore, a thrombosis model was established in zebrafish for the anti-thrombotic effect evaluation of DHI. We chose 3 dpf (days post fertilization) zebrafish as an appropriate stage of modeling, because zebrafish develop functional platelets and coagulation factors by 36 hpf (hours post fertilization) [35]. And for a better observation of tail and heart parts, 1-phenyl-2-thiourea (PTU) was utilized on the 24 hpf embryos as the melanogenesis inhibitor by blocking all tyrosinase-dependent steps in the melanin pathway. The concentration and initial time of PTU treatment was controlled to avoid toxicity and teratogenesis [36]. The model was developed by AA, since it could lead to the release of endogenous adenosine diphosphate (ADP) and induce the aggregation of blood platelets. Nevertheless, thromboxane A2 (TXA2) and prostacyclin (PGI2), a couple of metabolites of AA providing a balance control mechanism that intervenes thrombus and hemostatic plug formation, proved to be unequally produced and the metabolite of TXA2 was far more detected than the metabolite of PGI2, indicating the thrombosis-forming tendency after being treated with AA [37]. Aspirin was chosen as the positive drug of the AA-induced thrombosis, as reported, because it is an inhibitor of platelet cyclooxidase, which is an important participant of the AA cascade [38]. Since the zebrafish tail venous thrombus was reported to be reversely correlated with the RBC intensity [35], which could be positively correlated with the area of heart RBCs on the same direction. We chose the area of the heart red blood cells to represent the thrombus length/severity. The AA treated zebrafish showed a significant decrease of heart RBCs according to the images of the heart and the statistical results, along with severe aggregation of RBCs in the tail parts. And the slow flow of blood could be observed in a AA-treated transgenic LCR-GFP zebrafish on a video taken by a fluorescence microscope, additionally, suggesting that the model was successfully established. The damage caused by AA could be significantly improved by aspirin and DHI, respectively (Figure 1A,B). Nevertheless, we found that DHI is dose-dependent on the model in the range of 25–150 μL/mL, and it appears to have significant efficacy at a 75 μL/mL or higher dosage (Figure 2). In the acknowledgement that DHI was extracted from DS and HH, we investigated the anti-thrombotic effect of DS and HH by the model, respectively, in order to locate the Q-markers of DHI. As Figure 3 showed, both DS and HH showed excellent protection ability against thrombosis. According to the results reported by Liu and colleagues [29], six compounds belonging to DHI were picked, considering their content in DHI and activity, were investigated with the model we developed, respectively. The results illustrated that RA and LA contained in DS, and HSYA and pCA contained in HH, exhibited significant anti-thrombosis effects, which could thus be the candidates for the activity-based Q-markers. pCA was, for the first time, reported to be able to prevent thrombosis induced by AA. Thus, the quantitative assessment of pCA was carried out to explore the effective concentration. It suggested that pCA was dose-dependent at the range of 25–150 μg/mL and became effective at, or higher than, 50 μg/mL.

Furthermore, to determine if the compounds screened by the model were suitable for the Q-marker, and whether the model was qualified for ensuring the batch-to-batch efficacy of DHI, the discrimination of the compound content and anti-thrombotic effect between the normal DHI and abnormal DHI was detected by HPLC and the model, respectively. We prepared the abnormal samples mainly by ultraviolet radiation and heating reflux according to the reports that oxidation-induced degradation of salvianolic acid is the major cause of abnormal samples of DHI [39,40]. The abnormal DHI could be easily distinguished from the normal ones by the significant distinction of efficacy (represented by the heart RBCs showed on the model in Figure 6A). Thus, the batch-to-batch efficacy assurance could be realized in this manner. The abnormal DHI was validated by the content analysis, suggesting that the contents of major compounds, such as RA, pCA, and SaB, were decreased compared to those in the normal samples. Oppositely, the content of DSS was increased since it was the degradation product of SaB. According to the correlation coefficients between the anti-thrombotic effect and the contents of major compounds in DHI, the compound contents measured were closely correlated to the anti-thrombotic effect, except DSS. Among those compounds, RA and pCA had the potential to be regarded as representative Q-markers of DHI, since the SaB and DSS did not show significant anti-thrombotic effect according to the anti-thrombotic effect measured by the model. The ineffectiveness of abnormal DHI should be attributed to the rapid decrease of the content of RA and pCA, which showed superior ability in anti-thrombosis, and the increase of danshensu, which was not so active as RA and pCA after the modification of DHI. In accordance with the decrease of efficacy, which is represented by the inhibition rate (IR), the total content of RA and pCA proved to be significantly decreased. Thus, the RA and pCA can be regarded as two representative potential Q-markers of DHI, reflecting the anti-thrombotic effect.

In summary, the zebrafish thrombosis model proved to be suitable for the identification of Q-markers of DHI. Thus, the in vivo pharmacological models can be a potential tool in the application of quality assessment of CMs.

## 4. Materials and Methods

### 4.1. Chemicals, Drugs, and Reagents

Arachidonic acid (AA), dimethyl sulfoxide (DMSO), and *o*-dianisdine were purchased from Sigma-Aldrich Inc. (St. Louis, MO, USA). The salvianolic acid B (SaB), danshensu sodium (DSS), lithospermic acid (LA), rosmarinic acid (RA), p-coumaric acid (pCA), and hydroxysafflor yellow A (HSYA) were purchased from Shanghai Winherb Medical Technology Co., Ltd. (Shanghai, China). The aspirin was obtained from Dalian Meilun Biological Technology Co., Ltd. (Dalian, China). The 10 batches of normal danhong injection samples, the danshen component (DS), and the honghua component (HH) were provided by Danhong Pharmaceutical Co., Ltd. (Heze, China), while five batches of abnormal samples were collected from samples that suffered oxidative degradation. The glycerol and the 4% paraformaldehyde fix solution were purchased from Sangon Biotech Co., Ltd. (Shanghai, China). Ultrahigh-purity water was produced by a Millipore Milli-Q System (Milford, MA, USA). Acetonitrile, formic acid, and methanol were obtained from Merck KGaA (HPLC grade, Darmstadt, Germany). 1-phenyl-2-thiourea was purchased from Sigma-Aldrich Inc. (St. Louis, MO, USA).

### 4.2. Zebrafish Care and Maintenance

Zebrafish were bought from the China Zebrafish Resource Center (Wuhan, China). Adult wild-type Tuebingen (TU) strain zebrafish were used to complete all of the experiments, except the model validation, which was done by transgenic LCR-GFP zebrafish. All of the zebrafish were housed in a light- and temperature-controlled aquaculture facility with a standard 14 h:10 h light/dark photoperiod. They were fed with live brine shrimp twice daily and dry flake once a day. About five pairs of zebrafish were picked for natural mating every time, and about 300 embryos were generated on average. All of the embryos were maintained at 28.5 °C in fish water (containing 0.3% Instant Ocean Salt in deionized water with final pH 6.9–7.2, conductivity 500–550 μs/cm, and hardness of about 90 mg/L CaCO_3_). The embryos were picked and replaced with fresh fish water at 6 hpf (hours post fertilization) and 24 hpf to ensure the good condition of the embryos.

The transgenic LCR-GFP zebrafish were provided by Core Facilities, Zhejiang University School of Medicine (Hangzhou, China) and the method of maintenance was the same as the description above.

### 4.3. Experimental Protocol

The zebrafish were maintained in the fish water containing PTU at a final concentration of 2.5 μM during all of the experiments. The AA, aspirin, SaB, DSS, LA, RA, pCA, and HSYA were solved by DMSO to prepare stock solutions, and then further diluted to working solutions with PTU-containing fish water, while DHI, HH, and DS components were directly diluted by PTU-containing fish water to the working concentration. The final DMSO concentration was kept below 0.1%. The zebrafish larvae used in all of the experiments were 3 dpf. For all of the experiments, the zebrafish were exposed to the indicated concentration of drugs in 24-well plates, containing 11 fish per dish, two parallel dishes were set per group, and the temperature was set to 28.5 °C during all of the exposure times. The control group was treated with DMSO working solution containing the same concentration DMSO as other groups in the same experiment, and 40 μM AA was adopted for the modeling of thrombosis. The *o*-dianisidine was solved by absolute ethanol to the concentration of 5.85 mM as the stock solution, and was added by milliQ water (Milliport Corp., Billerica, MA, USA) 1 mL/mL, 0.1 M NaOAc (pH = 4.5, measured by a pH meter (Metter-Toledo International Inc., Zurich, Switzerland) 250 μL/mL, 30% H_2_O_2_ 50 μL/mL as the working solution.

### 4.4. Drug Efficiency Assessment

The 3 dpf zebrafish larvae were put into the 24-well plates. For the first six hours, the model group was treated with DMSO working solution equally as the control group. The tested groups were treated with test drugs at the indicated concentration to pre-protect. After incubation for six hours at 28.5 °C, all of the working solutions were replaced with fresh working solutions containing AA at the indicated concentration, except that the control group was replaced with fresh DMSO working solution, and the further incubation lasted one hour.

### 4.5. Staining and Fixing

After treatment, all of the incubation solutions were discarded and the zebrafish were transferred to 5 mL glass bottles and were stained with *o*-dianisidine working solution at 1 mL per bottle for 30 s without light. Then, the *o*-dianisidine working solution was abandoned and the zebrafish were washed by milliQ water three times. Then, the zebrafish were fixed in 4% paraformaldehyde for 10 h and were finally kept in 90% glycerol-water in milliQ water at 4 °C.

### 4.6. Image Capture and Quantitative Analysis of Zebrafish Thrombosis

For the quantitative analysis of the heart RBCs intensity, 10 zebrafish from each group were randomly picked for image capture of hearts and tails, respectively. All of the images were taken with identical lighting intensity at a 100× magnification with a dissecting stereomicroscope (Nikon Corporation, Tokyo, Japan) installed with a high-speed video camera (JVC). The image capture software was NIS Elements.D 4.30 (Nikon Corporation, Tokyo, Japan). The heart RBC intensity was quantitatively analyzed using Image-Pro Plus 6.0 (Media Cybernetios Inc., Rockvile, MD, USA) by the measurement of the area of heart RBCs, and the statistical analyses were performed by GraphPad Prism 6.01 (Graphpad Software Inc., La Jolla, CA, USA). One-way ANOVA was utilized to compare differences among groups, and *p* < 0.05 was considered statistically significant compared with the model group. The IC_50_ was calculated with GraphPad Prism 6.01. Furthermore, the statistical significance of the difference of efficacy between normal and abnormal DHI was investigated by the *t*-test at the 1% level (GraphPad Prism 6.01), and the inhibition rate (IR) was calculated with the following formulation [35]:

$$\mathrm{IR}=\frac{\left[E\left(\mathrm{drug}\right)-E\left(\mathrm{model}\right)\right]}{\left[E\left(\mathrm{control}\right)-E\left(\mathrm{model}\right)\right]}\times 100\%$$

As for the model validation, the transgenic zebrafish expressing the GFP reporter gene in mature erythrocytes [28] was adopted and images were taken at 100× magnification using a fluorescent microscope (Leica Microsystem Inc., Wetzlar, Germany) equipped with a camera. All images were taken under the same conditions, and the software used to capture the images was Andor SOLIS 4.27.30001.0 (Andor Technology Ltd., Belfast, UK).

### 4.7. Half-Quantitative Content Analysis of DHI

The 10 normal, and five abnormal, DHIs were half-quantitative content analyzed by HPLC on an Agilent 1100 Series HPLC system (Aglient Co. Ltd., Santa Clara, CA, USA) with a diode array detector using an Atiantis T3 column (250 mm × 4.6 mm, 5 μm, Waters Corporation, Milford, MA, USA). The temperature of the column was maintained at 25 °C. The UV detection wavelength was set at 280 nm for 0–75 min, and 326 nm for 75–130 min. The flow rate was 0.9 mL/min, and a gradient elution of mobile phase A (0.05% aqueous formic acid in water) and B (50% acetonitrile in water) was used. The gradient was started with 0% B, then was elevated to 18% B at 43 min, 25% B at 54 min, 38% B at 73 min, 48% B at 79 min, and kept at 48% B to 89 min, then to 60% B at 108 min, then to 100% B at 112 min, and kept at 100% B to 130 min. After that, the system was restored to the initial conditions in 10 min [41].

For the sample handling, 1 mL DHI samples of different batches (normal or abnormal) were accurately transferred to a 2 mL-volumetric flasks, respectively, and diluted with 20% methyl alcohol in water (containing 2% formic acid). Then the samples were centrifuged at a speed of 10,000 rpm for 10 min before injection for HPLC [41]. The abnormal samples were prepared by the following steps: Firstly, a series of normal samples were intensively mixed with air for about a week, and then they went through ultraviolet radiation for about three days and heating reflux at 70 °C for about three days.

The peaks of the HPLC chromatogram were identified using the method reported previously [41]. The peak areas were integrated, which could qualitatively represent the contents of the compounds by the Agilent ChemStation for LC Systems (Rev. B. 04. 03) (Aglient Co. Ltd., Santa Clara, CA, USA). Then, the distinction of each compound content between normal and abnormal samples was obtained accordingly. The correlation coefficients between the anti-thrombotic effect and the contents for major compounds in DHI were performed by SPSS 16.0 software (IBM Corporation, Amon, NY, USA). The SaB, DSS, RA, and pCA had been quantitatively assessed by the standard curve method, and the statistical significance of the difference of total content of RA and pCA between normal and abnormal DHI was investigated by the *t*-test at the 1% level (GraphPad Prism 6.01).

## 5. Conclusions

The zebrafish thrombosis model was utilized to validate the anti-thrombotic effect of DHI for the first time. The DHI and its components DS and HH, as well as several constituents, namely LA, RA, pCA, and HSYA, proved to possess significant anti-thrombotic effect. Combining the efficacy assessment and content analysis of normal and abnormal DHI, RA and pCA were identified as the efficacy-based potential Q-markers of DHI, and this model was considered to be appropriate for the quality assessment of DHI to ensure batch-to-batch pharmacodynamic consistency. Our findings provided a new strategy for the Q-marker identification of herbal medicine.

## Figures and Tables

**Table 1:** The correlation coefficients between the anti-thrombotic effect and the contents for major compounds in DHI.

	Correlation Coefficient
HMF *	0.9291
DSS	−0.7388
PA	0.8960
pCA	0.9150
SaD *	0.7826
RA	0.8564
SaB	0.7876
SaA *	0.9021

* HMF, SaD and SaA stand for 5-hydroxymethyl-2-furfural, salvianolic acid D and salvianolic acid A, respectively.
